# The Relationships between Empathy and Psychotic Experiences Along the Psychosis Continuum: Meta-analyses on Cognitive and Affective Empathy

**DOI:** 10.1192/j.eurpsy.2026.12122

**Published:** 2026-07-31

**Authors:** W. Yao, X. Sun, Q. Tang, K. H.-F. Cheng, A. K. C. Chau

**Affiliations:** 1 Department of Psychology, Hunan Normal University, Changsha, China; 2 Department of Psychology, Simon Fraser University, Vancouver, Canada; 3 Department of Psychiatry, The University of Hong Kong, Hong Kong, China

## Abstract

**Introduction:**

Deficits in cognitive empathy (CE) and affective empathy (AE) have been documented in patients with psychotic disorders and non-clinical people with psychotic experiences (PE). However, the associations of CE and AE with different dimensions of PE were inconsistently reported. Whether some PE dimensions are differentially associated with empathy, and whether these associations differ by participants’ characteristics (i.e., age, gender and clinical status) and measurement of empathy remain unclear.

**Objectives:**

To quantify the existing evidence for the associations of CE and AE with PE across the psychosis continuum using meta-analysis, and to compare them across PE dimensions, clinical status, age, gender and measurement of empathy.

**Methods:**

Following PRISMA guidelines, Medline, Web of Science, PsycInfo, PsycArticles and Embase were searched for records published up to March 2024. Studies published in English in peer-reviewed journals that reported correlations of CE or AE with PE in clinical or non-clinical samples were included. Three-level random-effects meta-analyses with restricted maximum likelihood estimation were performed.

**Results:**

Analysis of 317 effect sizes yielded a significant association between CE and PE (*r*=−0.13, *p*<.001). The negative dimension of PE (*r*=−0.174) showed a stronger association with CE than the positive dimension (*r*=−0.091), while the association with disorganized dimension was not significant (*r* =-0.096, *p*=0.878). Moreover, studies using behavioral tasks (*r*=−0.167) and clinical samples (*r*=−0.151) had stronger associations than those using self-report measures (*r=*-0.068) and non-clinical samples (*r=*-0.065), respectively. Studies with more females reported smaller effects (*r* =-0.077). Age had no significant moderating effect. Analysis based on 172 effect sizes revealed a non-significant association between AE and PE (*r*=−0.034, *p*=.375), with studies using behavioral tasks (*r*=−0.329) showing a stronger association than those using self-report measures (*r*=0.001). The moderating effects of clinical status, gender, and age were not significant.

**Conclusions:**

CE deficits were robustly linked to PE across the psychosis continuum, especially the negative dimension. This effect was stronger when using behavioral tasks, clinical samples and fewer female participants. Conversely, the pooled effect with AE deficits was only significant for studies using behavioral tasks. Interventions targeting CE may help preventing the development and maintenance of PE.

**Disclosure of Interest:**

None Declared

